# Epidemiological progression of COVID-19 positive cases among transnational truck drivers in the East African Region

**DOI:** 10.11604/pamj.supp.2022.41.2.29042

**Published:** 2022-03-29

**Authors:** Michela Martini, Ayomide Sina-Odunsi, Jaqueline Jael Dache, Julius Wekesa, Tasiana Mzozo

**Affiliations:** 1 International Organization for Migration (IOM), Regional Office for East and Horn of Africa, Nairobi, Kenya,; 2 World Health Organization, Kenya Country Office, Nairobi, Kenya

**Keywords:** COVID-19, truck drivers, epidemiology, East Africa

## Abstract

COVID-19 triggered a range of border controls to curb the spread of the disease. Containment measures and restrictions were put in place to mitigate cross border transmission while maintaining the flow of essential goods and services in the East and Horn of Africa Region. The first cases of COVID-19 detection among truck drivers, triggered and strengthened cross-border collaboration for detecting and responding to COVID-19 pandemic. Infection was significantly common among truck drivers in the region. As at 13 January 2021, there were 3,457 reported cumulative cases among truck drivers in the EHoA region. About 2,879 (83.3%) of the cases were reported in Uganda, 374 (10.8%) cases reported in Kenya, 190 (5.5%) cases reported in Rwanda and 14 (0.4%) cases reported in South Sudan. The reduction in the number of documented new COVID-19 cases among truck drivers declined with collaborative, timely and cooperative border point screening, and so preventing COVID-19 spread in the region. With most East African countries setting the stage for reopening borders and air spaces, sustained comprehensive surveillance is crucial for maintaining the gains from the collaborative response.

## Brief

COVID-19 had spread to all corners of the globe exacting a huge toll on health, social and economic systems with unceasing morbidities and mortalities. Africa remains vulnerable due to inadequate water sanitation and hygiene infrastructure, weak health systems, concurrent epidemics of vaccine-preventable and other infectious diseases, conflict and insecurity and population mobility. Governments in East and Horn of Africa (EHoA) have initiated timely various preparedness and response measures to contain or mitigate the spread of the pandemic. These measures include lockdown of non-essential travel in-country and between member states. Road transport remains a major conduit for delivery of cargo with transboundary travel cross-border, long distance truck drivers on a regular basis thereby increasing the risk of cross-border transmission of COVID-19. During the COVID-19 crisis, tens of thousands of truck drivers across 10 countries were able to maintain services to provide lifesaving and essential goods, including food, water, medicine, medical equipment and supplies, including supplies to meet the needs of humanitarian agencies assisting vulnerable communities such as Internally Displaced Persons (IDPs), stranded migrants [1] and other vulnerable population. While performing their work they have been expose to risk of COVID-19 more than others population and were the most frequently diagnosed category becoming in certain countries such as in Uganda [2] a priority group for COVID-19. The concerns of epidemic spread among this group is also linked to their social dynamics and interaction with community along transport corridors with the potential risk to generate significant local transmission. In addition, various mainstream media outlets have reported ongoing stigma and harassment that truck drivers experience at various border points, transport corridors and in the communities where they reside [3]. For the purpose of this brief we are presenting the epidemiological progression of COVID-19 positive cases among transnational truck drivers as reported by Ministry of health in affected countries namely Kenya, Rwanda, South Sudan and Uganda.

### Regional COVID-19 epidemiological trend among transnational truck drivers

**Figure 1:**
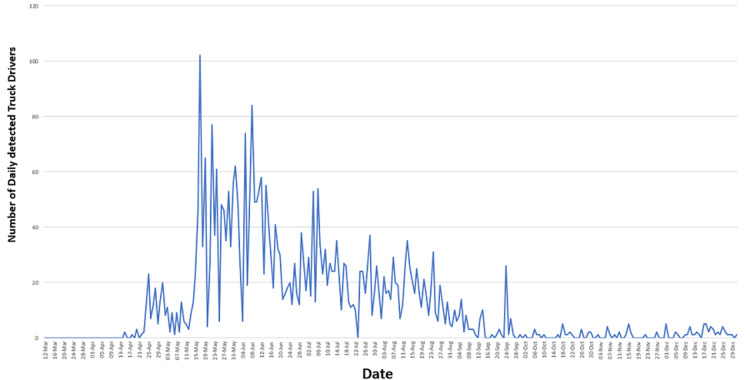
trend of new COVID-19 cases among truck drivers in East Africa

The first two COVID-19 positive cases among transnational cargo were confirmed on 14^th^ April 2020 at Malaba borders between Kenya and Uganda (one case Ugandan and One Kenyan) [4]. Since that, as at 13^th^ January 2021, there were 3,457 reported cumulative cases among truck drivers in the EHoA region. 2,879 (83.3%) of the cases were reported in Uganda, 374 (10.8%) cases reported in Kenya, 190 (5.5%) cases reported in Rwanda and 14 (0.4%) cases reported in South Sudan. This data needs to be read with attention to the fact that there is a discrepancy on reporting and testing among countries with Uganda the only country systematically reporting and sharing data (Figure 1).

The number of cases of truck drivers detected in the region steadily increased, with about 2-5 cases reported daily, and this prompted countries to increase testing capacities at border points. The increase in attention and number of tests to truck drivers led to more cases been detected. The trend continued to rise ranging between 50 and 100 cases daily from mid-May till the early August at most congested border points. Governments in the region ramped up COVID-19 prevention activities at the border points, this included mandatory pre-departure testing, testing at border points, relay trucking system and increased testing in at-risk cross border communities. A total of 1,054 cases was reported in June 2020, 121 cases in September 2020, 20 cases in November 2020 and 4 cases reported between 01^st^ January and 13^th^ January 2021. The epidemiological trend began to fall from August which may be attributed to the attention given to truck drivers across the region. The attention varies from one member country to the other.

### Uganda

**Figure 2:**
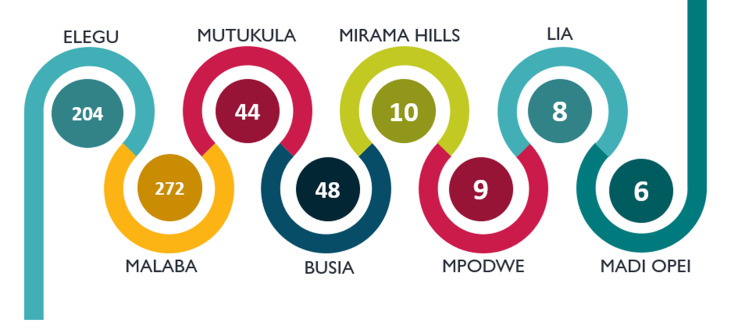
number of COVID-19 cases reported in Uganda at point of entry

The attention towards truck drivers coming into Uganda started when a 38-year-old Ugandan male, cargo transporter who arrived from Kenya via the Malaba border on 12^th^ April 2020 was tested positive to COVID-19 and a 27-year-old Kenyan truck driver who also arrived via the Malaba border on 14^th^ April 2020. As at 13^th^ January 2021, Uganda had reported a cumulative of 2,879 cases among truck drivers of which 747 are Ugandans. These cases have been identified from samples collected at border points for testing. About 6, 1, 2 and 204 cases were detected respectively at the Ugandan side of the Madi-Opei, Afoji, Kerwa and Elegu borders with South Sudan respectively; 272 and 48 cases were detected at Malaba and Busia borders with Kenya respectively; 44 cases reported at Mutukula border with Tanzania; 10 cases detected at Mirama Hills border with Rwanda; 6, 9 and 8 cases were detected at Pardewa, Mpondwe and Lia borders with DRC respectively (Figure 2).

On 16 May 2020, the number of cross-border truck drivers who had tested positive for COVID-19 stood at 143 out of the 203 confirmed COVID-19 cases in the country. The ministry of health announced a presidential directive stating that only truck drivers with negative test results for the virus will be allowed into the country. And since the directive has been given, 2,132 foreign truck drivers who tested positive for COVID-19 at border points were handed over to their country as at 28^th^ September 2020. These include 916 Kenyans, 255 Tanzanians, 68 Congolese, 30 Eritreans, 25 Burundians, 16 Rwandans, 10 South Sudanese, 3 Somali, 2 Egyptians and 1 Ethiopian (Table 1). There were also 806 more truck drivers recorded whose nationalities were not reported.

**Table 1:** number of truck drivers by Nationality who tested positive for COVID-19 at different border points in Uganda as at 28^th^ September 2020

Nationality	Number of cases detected
Kenya	916
Uganda	747
Tanzania	255
Congo	68
Eritrea	30
Burundi	25
Rwanda	16
South Sudan	10
Somalia	3
Egypt	2
Ethiopia	1
Nationality not Reported	806

### Kenya

**Figure 3:**
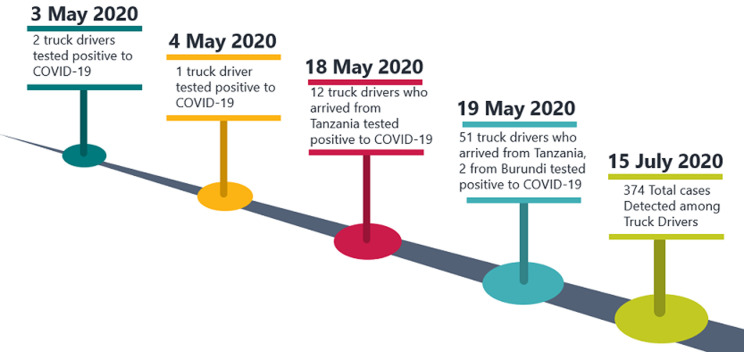
timeline of COVID-19 detected among truck drivers in Kenya

The cases of COVID-19 among cross border cargo transporters started on the 3^th^ May 2020 when 2 truck drivers were tested positive to COVID-19 and 1 case on 4 May, all of whom arrived from Uganda [5]. This increased the attention towards cross border infection. On the 18^th^ May 2020, 12 truck drivers all of whom arrived from Tanzania were tested positive and on the 19^th^ May, 51 who arrived from Tanzania and 2 who arrived from Burundi tested positive. The foreign truck drivers among the detected cases were all referred to their country of origin. As at 13^th^ January 2021, Kenya had reported 374 cases at the border points. At the Kenyan side of the Malaba and Busia borders with Uganda, 140 and 25 cases were detected respectively. Cases were also detected at Taita-Tateva border with Tanzania, and other border points (Figure 3).

### Rwanda

Rwanda started to identify an increase in COVID-19 cases among truck drivers at border points on the 24^th^ April 2020 when the first cases were reported among truck drivers from border points [6]. As of 13 January 2021, 190 cases have been recorded amongst Truck drivers, mainly at the Rusomo border with Tanzania and Rusizi Border with DRC as well as Nyagatare.

South Sudan started detecting cases of COVID-19 among truck drivers when 2 cases were detected on 28^th^ April 2020 at the Nimule border with Uganda [7]. Two (2) more cases were identified on the 1^st^ May 2020. Another truck driver detected on the 4^th^ May, 1 on the 5^th^ May, 2 cases on the 12^th^ May and 2 more cases on the 13^th^ May. As at 13^th^ January 2021, 14 cases of COVID-19 have been reported to be detected among truck drivers in South Sudan.

### Response

The countries swiftly instituted an effective health responses were put in place since the first COVID-19 cases detected among truck drivers. Ministries of health in affected countries put in place strict national preventive interventions including mandatory COVID-19 testing for truck drivers, Infection prevention Control (IPC) at borders and transport corridors. The COVID-19 tests created traffic congestion due to the long duration of the turnaround time. IOM in collaboration with Trademark East Africa (TMEA) supported the decongestion with emergency operation including deployment of medical staff and test equipment [8]. The East Africa Community (EAC) had issued immediate regional guidance [9] and practical tools such as the electronic tracking system for truck drivers [10]. This was to allow Member Countries enhance regional monitoring. WHO, IOM, UN agencies, EAC and Africa CDC had developed the regional strategy for management of COVID-19 along transport corridors to guide Member States in harmonized approach [11]. WHO and partners also facilitated cross-border coordination of the response efforts. For example, the recognition of COVID-19 test certificates, case management, infection prevention control measures and surveillance.

### Conclusion

There has been a significant reduction in the number of documented new COVID-19 cases among truck drivers, an indication that the timely and collaborative interventions taken by Ministry of Health, EAC and UN agencies and other partners were effective. Road transport is a significant conduit for cargo delivery across boundaries and truck drivers increased the risk of COVID-19 cross-border transmission. Although East African countries have reopened their air spaces, the transnational truck drivers remain a significant high-risk group in the region and the comprehensive surveillance should be sustained.

### Limitations

Findings of our communication must be considered in view of its limitations. Firstly, the data used is based on official reporting from Ministries of Health and it is important to note that there is a lack of reported data in some countries over a period of time. Since 20^th^ June 2020, only the Ugandan Ministry of Health had continued systematic reporting on new cases detected among truck drivers. Also, there is a lack of information on number of tests conducted on truck drivers making it difficult to have reliable data on positivity rate. However as for official data we can confirm a decline of incidence cases among the sample study.
